# Impact of performance-based financing on maternal and child health outcomes at the subnational level in the Central African Republic

**DOI:** 10.11604/pamj.2025.52.14.48186

**Published:** 2025-09-11

**Authors:** Ngouyombo Ange Donatien, Woromogo Sylvain Honoré, Doyama-Woza Rodrigue Herman, Garoua-Adjou Stéphanie Inesse, Folefack Tatsadong Christelle Corinne, Longo Jean de Dieu

**Affiliations:** 1 Inter-State Center for Higher Education in Public Health of Central Africa, Bangui, Central African Republic,; 2 Doctoral School of Human and Veterinary Sciences, University of Bangui, Bangui, Central African Republic,; 3 Department of Public Health, Faculty of Health Sciences, University of Bangui, Bangui, Central African Republic,; 4 Ministry of Public Health and Population, Bangui, Central African Republic

**Keywords:** Maternal and child health, performance-based financing, Central African Republic

## Abstract

**Introduction:**

maternal and child health remains a major challenge in the Central African Republic (CAR), with alarming indicators worsened by recurrent crises. This study aims to assess the impact of performance-based financing (PBF), implemented as part of the Health System Support Project (PASS), on maternal and child health indicators in the Kémo health district between 2019 and 2020. The study aims to assess the impact of PBF on maternal and child health indicators.

**Methods:**

this retrospective cross-sectional study with a before/after comparison was conducted in June 2024 in 14 health facilities in the sub-prefectures of Dekoa and Mala (Kémo health district, CAR), which had implemented performance-based financing between 2019 and 2020. Convenience sampling was used. The analysis was carried out using Excel and Epi-info version 7.2.6.

**Results:**

the study revealed that the number of skilled personnel almost doubled in all professional categories after the introduction of PBF. A significant improvement in medical equipment and health infrastructure was noted. Coverage of delivery assisted by skilled personnel increased from 64.76% before to 71.95% after, OR = 1.39; 95% CI = [1.27-1]. Coverage of ANC4+ antenatal consultation increased from 58.90% before to 93.58% after, OR= 10.17 95% CI= [9.55-10.84]. Coverage of Penta 3 vaccine increased from 48.30% before to 79.57% after, OR = 4.17; 95% CI= [3.18-4.48]. All these results were significant, with p < 0.05.

**Conclusion:**

performance-based financing has improved maternal and child health indicators in the district. As for its effect on improving the quality of maternal and child health care, other factors need to be taken into account, which will require further study.

## Introduction

Maternal, newborn and child health (MNCH) is a public health priority, encompassing all medical, social and educational interventions aimed at ensuring the physical, mental and social well-being of women during pregnancy, childbirth and the postnatal period, as well as that of newborns and children up to the age of five [1]. It includes prenatal and postnatal care, family planning, nutrition, vaccination, prevention of childhood diseases, monitoring of child development and health education for families. This integrated approach aims to reduce maternal and infant morbidity and mortality [2,3]. In the Central African Republic (CAR), maternal, newborn, and child health (MNCH) remains a major challenge, with particularly worrying health indicators. In 2017, the maternal mortality rate was estimated at 917 deaths per 100,000 live births, while infant mortality reached 86.32 deaths per 1,000 live births. These figures remain well above the targets set by the Sustainable Development Goals (SDGs), which aim for fewer than 70 maternal deaths and 25 infant deaths, respectively, for the same ratios [4,5]. Recurring military crises have seriously compromised the health system, leading to vandalism of health infrastructure, attacks on health personnel, and the abandonment of services by many qualified professionals. This situation increases the vulnerability of women and children and calls for an urgent and coordinated response [6].

Faced with this situation, the Central African government, with the support of the World Bank, launched the Health System Support Project (PASS). This project aims to strengthen primary health care through a performance-based financing (PBF) approach, which makes payments conditional on the achievement of measurable results. The PASS primarily targets pregnant women, children under five, and vulnerable populations, including indigenous communities [7]. The Kémo health district was selected for the pilot implementation of the Health System Support Program (PASS) due to the weakness of its maternal, newborn, and child health (MNCH) indicators prior to the intervention. Vaccination coverage for the Penta 3 vaccine was less than 50%, an assisted delivery rate of around 65%, and severely degraded health infrastructure was observed. This context represented a strategic opportunity to test the effectiveness of performance-based financing (PBF) in a post-crisis environment [8]. The PBF approach has already shown promising results in several countries in the region. In Burundi, it has led to a significant improvement in the coverage of essential health services [9]. In Cameroon, it has increased the availability of medical equipment and staff motivation [10]. In Mali, it has improved prenatal and postnatal consultations [11]. This study aims to assess the impact of performance-based financing (PBF), implemented as part of the Health System Support Project (PASS), on maternal and child health indicators in the Kémo health district between 2019 and 2020 in order to answer the following question: has PBF improved the coverage of maternal and child health services?

## Methods

**Study design:** this is a retrospective cross-sectional study with a before/after comparison, conducted in the Kémo health district (RCA) from 1^st^ June to 30^th^ June, 2024. It analyzes maternal and child health data for the period 2017-2020. The “before” phase (2017-2018) precedes the introduction of performance-based financing (PBF), implemented in 2019, while the “after” phase covers 2019-2020.

**Study framework:** the Kémo health district, located in Health Region No. 4 of the Central African Republic, covers four sub-prefectures: Sibut, Dekoa, Mala, and Ndjoukou. It includes 36 health facilities (FOSA), comprising a district hospital, a secondary hospital, health centers, and health posts, which provide services according to their level: minimum package of activities (MPA), complete package of activities (CPA). The district presents geographic and demographic diversity, with unequal access to healthcare. All FOSA received support through the Health System Support Project (PASS), funded by the World Bank, via performance-based financing (PBF). Some health facilities also benefited from additional support from technical and financial partners in a post-conflict context. Dekoa and Mala were selected for this study due to their semi-urban and rural profiles and the absence of external support beyond performance-based financing, allowing for an isolated assessment of their impact.

**Duration of study:** the study was conducted from 1^st^ to 30^th^ June 2024.

**Study period:** the study period was retrospective over 4 years, from 1^st^ January 2017 to 31^st^ December 2020.

**Study participants:** the study participants consist of health facilities that implemented performance-based financing (PBF) in the sub-prefectures of Dekoa and Mala between 2019 and 2020. These structures represent the main units of analysis, due to their direct involvement in the PASS project. The data used come from institutional databases, notably District Health Information Software 2 (DHIS2), which centralize information relating to performance indicators, resource management and the quality of services offered.

**Inclusion criteria:** the study included health facilities located in the Kémo health district, specifically in the sub-prefectures of Dekoa and Mala, which implemented Performance-Based Financing (PBF) between 2019 and 2020. The selection concerned facilities with complete data on maternal and child health indicators.

**Exclusion criteria:** health facilities not participating in the implementation of performance-based financing (PBF) during the period under review were excluded from the study. Similarly, facilities with incomplete, missing, or unusable data on maternal and child health indicators were excluded.

**Sampling type:** a convenience sampling was conducted, including all health facilities located in the sub-prefectures of Dekoa and Mala that met the inclusion criteria defined for this study.

**Sample size:** the sample size, consisting of 14 health facilities, was determined by purposive sampling. All facilities that implemented performance-based financing (PBF) in the sub-prefectures of Dekoa and Mala between 2019 and 2020 were included. This selection was based on their direct participation in the PASS project, the absence of external support beyond performance-based financing, as well as the availability of their data in District Health Information Software 2 (DHIS2). This choice aimed to ensure full representativeness of the facilities involved in the intervention in the study area.

### Data sources and variables

**Variables:** the study compared the following indicators before and after the implementation of performance-based financing (PBF): i) Human resources: number and categories of qualified personnel. ii) Material and infrastructure resources: number of material and infrastructure. iii) Maternal and child health indicators, including (births assisted by qualified personnel, prenatal care (≥ 4 visits, CPN4+), postnatal consultations (CPoN), cesarean section coverage, curative consultations for children under 5 and over 5 years old, minor surgeries, tetanus vaccination (VAT2+), pentavalent vaccine (third dose, Penta3), family planning coverage). iv) The dependent variables in this study were key maternal and child health indicators, including assisted deliveries, antenatal consultations (≥4 visits), postnatal consultations, caesarean sections, curative consultations for children under five, minor surgeries, and immunization coverage (VAT2+, Penta 3). The independent variable was the implementation of performance-based financing (PBF), categorized as “Yes” for the period 2019-2020 and “No” for the period 2017-2018.

**Data collection:** the data were extracted using standardized documentary review forms from the databases of the National Health Information System (NHIS) of the Ministry of Health of the CAR, through the District Health Information Software 2 (DHIS2).

**Data analysis:** data entry and analysis were performed using Excel and Epi Info version 7.2.6 software. Performance indicators were compared between two periods: before the intervention (2017-2018) and after the intervention (2019-2020). For each indicator, the proportions of targets achieved and not achieved were calculated. Data were analyzed using binary logistic regression to estimate the association between PBF implementation and the achievement of maternal and child health targets. Odds ratios (OR) with 95% confidence intervals (CI) were calculated for each indicator. A p-value < 0.05 was considered statistically significant. The independent variable was the presence of performance-based financing (PBF), coded as “1” for the 2019-2020 period (with PBF) and “0” for the 2017-2018 period (without PBF). The dependent variables were dichotomous.

**Ethical consideration:** the study was conducted in accordance with the ethical principles of the Declaration of Helsinki. Ethical approval was granted by the Scientific and Ethics Committee for Research of the *Université de la Montagne* (CSERC/UdM) under reference number 0011/CSERC/UdM/2024. Administrative authorization was also granted by the Ministry of Public Health of the Central African Republic.

## Results

**Human resources situation before and after performance-based financing implementation (2017-2020):** the number of doctors increased from 2 to 5, an increase of 3 people. For the IDE/SF/TSL (State Registered Nurses/Midwives/ Senior Laboratory Technicians) category, the number more than doubled, from 8 to 18. The AS/AA (Health Assistant/Midwife Assistant) almost tripled, from 8 to 22. The number of cleaners and guards increased slightly, from 47 to 51, an increase of 4 people. However, the number of IS/MA (First Aid Nurse/Midwife Matron) remained constant at 79 (Table 1).

**Table 1:** evolution of health personnel numbers in the 14 health facilities of the Dekoa and Mala sub-prefectures (Kémo Health District), before (2017-2018) and after (2019-2020) the implementation of PBF

Categories	Health personnel before FBP (2017-2018)	Health personnel before FBP (2019-2020)	Variation
Doctors	2	5	3
IDE/SF/TSL	8	18	10
AS/AA	8	22	14
IS/MA	79	79	0
Surface technicians /guardians	47	51	4
TOTAL	**144**	**175**	**31**

IDE/SF/TSL: state registered nurse/midwife/Senior laboratory technician**;** AS/AA: Health assistants/Midwife assistants**;** IS/MA: First aid nurse/Midwife; FBP: foreign body response; PBF: performance-based financing

**Material resource situation before and after performance-based financing implementation (2017-2020):** the total number of delivery tables increased from 12 to 23. The number of consultation tables increased from 14 to 35. The total number of hospital beds increased from 67 to 182. The number of minor surgery boxes doubled from 23 to 55. The number of oxygen concentrators increased from 1 to 7. We noted the acquisition of 2 ambulances. The number of motorcycles increased from 6 to 18 (Table 2).

**Table 2:** situation of material resources in the 14 health facilities of the Dekoa and Mala sub-prefectures (Kémo Health District), before (2017-2018) and after (2019-2020) the implementation of PBF

Materials	Material resources before foreign body response (2017-2018)	Material resources after foreign body response (2019-2020)	Variation
Delivery tables	12	23	11
Look-up table	14	35	21
Operating table	01	03	2
Hospital bed	67	182	115
Delivery box	25	49	24
Caesarean section box	04	08	4
Minor surgery box	23	55	32
Oxygen concentrator	01	07	6
Vaccine refrigerator	06	15	9
Ambulance	00	02	2
Motorcycle	06	18	12

**Health infrastructure situation before and after performance-based financing implementation (2017-2020):** regarding health infrastructure, maternity wards were renovated or improved, increasing from 0 to 12 in good condition. Similarly, operating theaters were upgraded, increasing from 0 to 12 in good condition. Laboratories also benefited from renovations, increasing from 0 to 2 in good condition. The number of hospitalization and medical observation rooms increased from 39 to 58, with an improvement in their condition, increasing from 11 to 58 in good condition. Finally, post-surgical follow-up rooms were reinforced, increasing from 8 to 16, and their condition improved, increasing from 3 to 16 in good condition (Table 3).

**Table 3:** situation of health infrastructure in the 14 health facilities of the Dekoa and Mala sub-prefectures (Kémo Health District), before (2017-2018) and after (2019-2020) the implementation of PBF

Structures	Health infrastructure before FBP ( 2017-2018)		Health infrastructure after FBP (2019-2020 )	
	Existence	Good condition	Existence	Good condition
Maternity	12	0	12	12
Operating room	2	0	2	2
Laboratory	3	0	3	2
Hospitalization room/Medical observation	39	11	58	58
Post-surgical monitoring room	8	3	16	16

**Coverage of maternal and child health indicators before and after performance-based financing implementation (2017-2020):** coverage of births attended by qualified personnel increased from 64.76% to 70.53%. Prenatal consultations (at least four) increased slightly, from 58.90% to 59.29%. Postnatal consultations increased from 29.43% to 48.17%. Coverage of caesarean sections increased from 40.33% to 53.40%. Curative consultations for children under five years of age increased from 68.82% to 77.34%, and for those five years of age and older, from 35.34% to 63.82%. Coverage of minor surgeries increased from 52.15% to 59.76%. Tetanus vaccination among women of childbearing age increased from 28.54% to 50.45%, while the pentavalent (Penta3) vaccine increased from 48.30% to 79.57%. Finally, family planning availability increased from 69.08% to 88.21% (Table 4).

**Table 4:** evolution of coverage of maternal and child health indicators in the 14 health facilities of the Dekoa and Mala sub-prefectures (Kémo Health District), before (2017-2018) and after (2019-2020) the implementation of PBF

Indicators	Coverage of indicators before FBR (2017-2018)			Coverage of indicators after FBR (2019-2020)		
	N	n	%	N	n	%
Childbirth	3768	2440	64.76	3960	2849	71.95
CPN4+	28128	16566	58.90	29556	27659	93.58
CPoN	12480	3673	29.43	13200	6359	48.17
Caesarean sections	300	121	40.33	324	173	53.40
Consultations - 5 years	47892	32957	68.82	50304	38907	77.34
Consultation 5 years and over	116916	41316	35.34	122832	78389	63.82
Minor surgeries	7584	3955	52.15	7980	4769	59.76
VAT2+	16548	4722	28.54	17376	8966	51.60
Penta 3	7392	3570	48.30	7752	6168	79.57
Family Planning	2532	1749	69.08	2664	2350	88.21

N=expected targets for the year, n=number achieved, CPN4+ 4 prenatal consultations and more CPoN postnatal consultation, VAT2= Tetanus vaccination; FBR: foreign body response

**Coverage of maternal and child health indicators before and after performance-based financing implementation (2017-2020):** coverage of births attended by qualified personnel increased from 64.76% to 70.53%. Prenatal consultations (at least four) increased slightly, from 58.90% to 59.29%. Postnatal consultations increased from 29.43% to 48.17%. Coverage of caesarean sections increased from 40.33% to 53.40%. Curative consultations for children under five years of age increased from 68.82% to 77.34%, and for those five years of age and older, from 35.34% to 63.82%. Coverage of minor surgeries increased from 52.15% to 59.76%. Tetanus vaccination among women of childbearing age increased from 28.54% to 50.45%, while the pentavalent (Penta3) vaccine increased from 48.30% to 79.57%. Finally, family planning availability increased from 69.08% to 88.21% (Table 4).

**Association between performance-based financing and changes in maternal and child health indicators:** statistical analysis revealed a significant association between the implementation of performance-based financing (PBF) and the improvement of the indicators studied. Women were more likely to deliver in health facilities (OR = 1.39) and to attend postnatal consultations (OR = 2.23). The likelihood of attending at least four antenatal consultations increased tenfold (OR= 10.17). Caesarean section coverage also improved (OR= 1.69). Among children, curative consultations increased for those under five years (OR= 1.55) and for those aged five and above (OR= 3.22). Tetanus vaccination coverage among women of reproductive age (OR= 2.67) and pentavalent vaccine coverage among children (OR= 4.17) showed significant improvement. Access to family planning services tripled (OR= 3.35), and minor surgical procedures also increased (OR= 1.52). All these results are statistically significant with p < 0.001 (Table 5).

**Table 5:** research on the association between FBR and the evolution of maternal and child health indicators in the 14 health facilities of the Dekoa and Mala sub-prefectures (Kémo Health District)

FBR	Targets achieved		Targets not met		OR (95% CI)	P value
	n	%	n	%		
	**Births assisted by qualified personnel**					
Yes (2019-2020)	2849	71.95	1167	28.05	1.39 (1.27-1.53)	< 0.001
No (2017-2018)	2440	64.76	1328	35.24		
	**Pregnant women who have had more than 4 CPNs**					
Yes (2019-2020)	27659	93.58	1202	06.42	10.17 (9.55-10.84)	< 0.001
No (2017-2018)	16566	58.90	11562	41.10		
	**Postnatal consultations**					
Yes (2019-2020)	6359	48.17	6841	51.83	2.23 (2.12-2.35)	< 0.001
No (2017-2018)	3673	29.43	8807	70.57		
	**Caesarean sections**					
Yes (2019-2020)	173	53.40	151	46.60	1.69 (1.23-2.33)	< 0.001
No (2017-2018)	121	40.33	179	59.67		
	**Curative consultations for children under 5 years old**					
Yes (2019-2020)	38907	77.34	11397	22.66	1.55 (1.50-1.59)	< 0.001
No (2017-2018)	32957	68.82	14935	31.18		
	**Minor surgeries in children**					
Yes (2019-2020)	4769	59.76	3211	40.24	1.36 (1.28- 1.50)	< 0.001
No (2017-2018)	3955	52.15	3629	47.85		
	**VAT2+**					
Yes (2019-2020)	8966	51.60	8410	48.40	2.67 (2.55 – 2.79)	< 0.001
No (2017-2018)	4722	28.54	11826	71.46		
	**Penta 3**					
Yes (2019-2020)	6168	79.57	1584	20.43	4.17 (3.18–4.48)	< 0.001
No (2017-2018)	3570	48.30	3822	51.70		
	**Consultation 5 years and over**					
Yes (2019-2020)	78389	63.80	44483	36.20	3.22 (3.17 – 3.28)	< 0.001
No (2017-2018)	41316	35.34	75600	64.66		
	**Family planning**					
Yes (2019-2020)	2350	88.21	314	11.79	3.35 (2.89 – 3.87)	< 0.001
No (2017-2018)	1749	69.08	783	30.92		

CPN4+ = 4 prenatal consultations plus, CPoN = postnatal consultation, targets reached = Number of people who benefited from the procedure, targets not reached = Number of people who did not benefit from the procedure; FBR: foreign body response

## Discussion

**Human resources situation before and after performance-based financing implementation (2017-2020):** data analysis reveals a significant improvement in the availability of qualified human resources in health facilities after the implementation of performance-based financing (PBF). Compared with the period before the introduction of PBF, the number of qualified personnel almost doubled in all categories. This finding corroborates the results observed during the PBF pilot phase in Mali, where Gautier *et al*. noted an increase in the number of qualified health workers, linked to recruitments carried out by the government [12]. In our study, this increase could be explained by recruitments carried out directly by health facilities, in accordance with the planning included in their business plans, developed with the support of the Ministry of Health and Population and the PBF implementation unit.

**Material resources situation before and after performance-based financing implementation (2017-2020):** our study reveals a significant improvement in material resources in health facilities after the introduction of performance-based financing (PBF). This improvement is manifested by an increase in the number of available equipment and the acquisition of new biomedical materials. This finding is consistent with the results of the study conducted by Hamadou Saidou in Cameroon, where the proportion of health centers with appropriate equipment increased from 63% to 77% after the implementation of PBF [10]. This positive development can be attributed to the implementation of a subsidy distribution key by the Ministry of Health and Population, which allocates 20% of the funds generated by the FOSA to investment, particularly for the purchase of equipment and the improvement of the technical platform. This mechanism allows health structures to plan their equipment needs as part of their business plan, thus promoting more autonomous and strategic management of resources.

**Health infrastructure situation before and after performance-based financing implementation (2017-2020):** our study highlights a significant improvement in health infrastructure following the implementation of performance-based financing (PBF). This improvement resulted in the rehabilitation and construction of several rooms in targeted health facilities. This finding is consistent with observations made in other African contexts, where PBF has enabled health facilities to strengthen their technical platform thanks to greater management autonomy and targeted financial incentives [13]. Similar results were observed in Ethiopia and Zimbabwe, where PBF was institutionalized and integrated into national health policies. These countries saw significant improvements in the quality of care through infrastructure investments, particularly in remote rural areas [14]. These findings confirm that PBF, when well-structured and accompanied by a clear governance framework, can effectively contribute to the modernization of health infrastructure, provided that monitoring and maintenance mechanisms are also strengthened.

**Coverage of maternal and child health indicators before and after performance-based financing implementation (2017-2020):** before the introduction of performance based financing (PBF), the coverage of births attended by skilled personnel was 64.76%. After its implementation, it reached 71.95%, an increase of 7.19%. This coverage remains lower than those obtained in Burkina Faso in 2020, Bamako in 2018, Burundi which are respectively 77.2%, 35.5% and 18% [9,15,16]. Conversely, Mali experienced a decrease, going from 64.6% to 58.8%, a decrease of 5.8% [17]. The improvement in the Central African Republic (CAR) could be explained by the recruitment of qualified personnel, the improvement of health infrastructure, but also by the establishment of an incentive mechanism encouraging traditional midwives to refer women to maternity wards. Furthermore, the coverage of women having had at least four prenatal consultations (CPN4) increased from 58.90% to 93.58%, an increase of 34.68%. This increase is higher than that observed by Traoré Bakari in Mali, which is 21.2% [11]. The improvement observed in our study can be attributed to the availability of qualified personnel, the reduction in costs associated with antenatal care, as well as community mobilization facilitated by the introduction of performance-based financing. Regarding postnatal consultations (CPoN), coverage increased from 29.43% to 48.17%, an improvement of 18.74%. This increase is higher than that observed by Traoré Bakari in Mali, which is 7.29 [11]. This improvement can be attributed to several factors, including the active involvement of midwives in rural maternity wards in the provision of postnatal consultation services (CPON), as well as the participation of community health workers and community relays in awareness-raising activities for women in the postpartum period, particularly during home visits for child monitoring. Regarding the coverage of caesarean sections, it increased from 40.33% to 53.40%, an increase of 13.07%. Although this improvement is slightly lower than that observed in Burundi (16%) [9]. It could be linked to an upward revision of reimbursement for caesareans under the FBP, thus facilitating access to this intervention. However, this also raises the question of a possible excessive use of this practice.

For children under five, the coverage of curative consultations increased from 68.82% to 77.34%, an increase of 8.52%. However, this increase remains lower than that reported by Yohou in Côte d'Ivoire (64.9%) and by Coulibaly in Bamako, Mali (34%) [18,19]. As for children aged five and over, consultation coverage increased from 35.34% to 63.80%, an increase of 28.46%. Despite this, this improvement remains lower than that of Burundi, which is 54% [9]. This could be explained by the fragility of the Central African health system, weakened by repeated crises. Thanks to PBF, qualified human resources have been recruited, medicines have become more available, and infrastructure has been strengthened. At the same time, vaccination coverage for the third dose of the pentavalent vaccine (Penta 3) increased from 48.30% to 79.57%, an increase of 31.27%. This increase is much higher than that observed in Burundi (9%) [9]. In the Central African Republic, all health facilities are mandated to vaccinate newborns. However, prior to the implementation of performance-based financing (PBF), many lacked the necessary logistical and operational resources to do so effectively. The introduction of PBF enabled the acquisition of motorcycles by health facilities, facilitating outreach strategies in remote areas. Additionally, both passive and active cold chain equipment were provided, and staff motivation was enhanced through financial incentives. These combined efforts contributed substantially to the improvement of vaccination indicators across the district. Finally, family planning coverage improved from 69.08% to 88.21%, an increase of 19.13%, which is higher than that observed by Traoré Bakari in Mali, which is 4.6% [11]. This increase would be due to the availability of family planning inputs facilitated by the FBP project.

**Association between performance-based financing and changes in maternal and child health indicators:** in analyzing the results of our study, we found that performance-based financing (PBF) had a significant influence on the ten maternal and child health indicators studied, with a p-value < 0.05. However, in Burkina Faso, some authors observed that, among the five maternal and child health indicators studied, only the coverage of postnatal consultations in district hospitals benefiting from PBF was significantly higher, with a value of P= 0.04. This does not clearly demonstrate the effectiveness of PBF in improving maternal and child health indicators in Burkina Faso [20]. In the Central African Republic (CAR), the health system has been weakened by multiple crises, resulting in poor maternal and child health indicators. The introduction of PBF would have strengthened the health system through the recruitment of qualified personnel, improvement of infrastructure and technical facilities, staff motivation, availability of medicines and inputs, community awareness raising and subsidies for indicators that have reduced the cost of services, thus making services more accessible.

**Limitations of the study:** this study presents certain limitations: i) lack of control for confounding factors. ii) risk of overreporting performance indicators. iii) absence of sensitivity or subgroup analyses.

## Conclusion

The implementation of performance-based financing (PBF) in the sub-prefectures of Dekoa and Mala in the Central African Republic led to improvements in several maternal and child health indicators between 2019 and 2020. Significant progress was observed in human resources, medical equipment, health infrastructure, and the coverage of key services such as antenatal consultations (ANC4+), Penta 3 vaccination, and family planning. However, not all indicators showed uniform progress. Some, such as assisted deliveries and curative consultations for children, demonstrated only modest improvements, suggesting disparities in the effectiveness of PBF depending on local contexts. This heterogeneity calls for a more in-depth analysis of the existing dataset to identify limiting factors and specific levers for each indicator. Statistical analysis revealed a significant association between the implementation of performance-based financing (PBF) and the positive evolution of the indicators studied, with p-values less than 0.05. Although these results are encouraging, they should be interpreted with caution due to several limitations. These include the lack of control for confounding factors, the risk of overreporting performance indicators due to financial incentives, and the absence of sensitivity or subgroup analyses. Nevertheless, these findings provide a solid foundation for guiding health policies, strengthening monitoring mechanisms, and further evaluating the impact of PBF on the quality of care. Thus, this study provides useful elements to decision-makers and technical partners to improve the implementation of PBF and adapt health system strengthening strategies in fragile contexts. In addition, we recommend conducting a complementary qualitative study involving healthcare providers, facility managers, and beneficiaries to better understand the internal dynamics, perceptions, and organizational or community-level barriers that influence outcomes. Such an approach would enrich the interpretation of quantitative data and help refine strategies to improve PBF implementation in post-conflict settings.

### What is known about this topic

- Performance-Based Financing (PBF) is a strategy that has been implemented in several sub-Saharan African countries, including Burundi, Mali, Cameroon, Rwanda and Democratic Republic of the Congo; it aims to improve access to and the quality of healthcare services, particularly in maternal and childhealth;
- Performance-based financing is based on conditional financial incentives, which are granted upon the achievement of measurable targets; rigorous verification mechanisms are used to ensure the quality of services delivered.

### What this study adds

- Following the introduction of PBF, there has been a notable strengthening of both human and material resources; the number of qualified health workers has nearly doubled, medical equipment has been significantly improved, and several key infrastructures including maternity wards, laboratories, and hospitalization rooms have been either renovated or newly constructed; we also observed a steady improvement in maternal and child health indicators.
